# HashClone: a new tool to quantify the minimal residual disease in B-cell lymphoma from deep sequencing data

**DOI:** 10.1186/s12859-017-1923-2

**Published:** 2017-11-23

**Authors:** Marco Beccuti, Elisa Genuardi, Greta Romano, Luigia Monitillo, Daniela Barbero, Mario Boccadoro, Marco Ladetto, Raffaele Calogero, Simone Ferrero, Francesca Cordero

**Affiliations:** 1 Department of Computer Science, University of Torino, Via Pesinetto 12, Turin, 10149 Italy; 2 Division of Hematology, Department of Molecular Biotechnologies and Health Sciences, University of Torino, Via Genova 3, Turin, 10126 Italy; 3 Division of Hematology, Az. Ospedaliera SS Antonio e Biagio e Cesare Arrigo, Via Venezia 16, Alessandria, 15121 Italy; 4 Department of Molecular Biotechnology and Health Sciences, University of Torino, Via Nizza 52, Turin, 10125 Italy

**Keywords:** Clonality assessment, Minimal residual disease monitoring, Hash-based algorithm

## Abstract

**Background:**

Mantle Cell Lymphoma (MCL) is a B cell aggressive neoplasia accounting for about the 6% of all lymphomas. The most common molecular marker of clonality in MCL, as in other B lymphoproliferative disorders, is the ImmunoGlobulin Heavy chain (IGH) rearrangement, occurring in B-lymphocytes. The patient-specific IGH rearrangement is extensively used to monitor the Minimal Residual Disease (MRD) after treatment through the standardized Allele-Specific Oligonucleotides Quantitative Polymerase Chain Reaction based technique. Recently, several studies have suggested that the IGH monitoring through deep sequencing techniques can produce not only comparable results to Polymerase Chain Reaction-based methods, but also might overcome the classical technique in terms of feasibility and sensitivity. However, no standard bioinformatics tool is available at the moment for data analysis in this context.

**Results:**

In this paper we present HashClone, an easy-to-use and reliable bioinformatics tool that provides B-cells clonality assessment and MRD monitoring over time analyzing data from Next-Generation Sequencing (NGS) technique. The HashClone strategy-based is composed of three steps: the first and second steps implement an alignment-free prediction method that identifies a set of putative clones belonging to the repertoire of the patient under study. In the third step the IGH variable region, diversity region, and joining region identification is obtained by the alignment of rearrangements with respect to the international ImMunoGenetics information system database. Moreover, a provided graphical user interface for HashClone execution and clonality visualization over time facilitate the tool use and the results interpretation. The HashClone performance was tested on the NGS data derived from MCL patients to assess the major B-cell clone in the diagnostic samples and to monitor the MRD in the real and artificial follow up samples.

**Conclusions:**

Our experiments show that in all the experimental settings, HashClone was able to correctly detect the major B-cell clones and to precisely follow them in several samples showing better accuracy than the state-of-art tool.

**Electronic supplementary material:**

The online version of this article (doi:10.1186/s12859-017-1923-2) contains supplementary material, which is available to authorized users.

## Background

In the last years, the introduction of new drugs and therapeutic schedules have improved the clinical outcome of patients affected by hematologic disease, especially in B-cell lymphoma [1]. Despite the significant therapeutic progresses reached, several patients still relapse and die due to the emergence of resistant new clones. Based on these reasons, molecular markers detection at diagnosis and early identification of patients at high risk of relapse during the natural history of the disease are the major objectives of current onco-hematology translational research. Therefore, a relevant challenge is to support the clinical therapeutic decisions through the identification and the monitoring of the clonal subpopulations in a prospective way, using methods that quantify residual tumour cells beyond the sensitivity level of routine imaging and laboratory techniques [2].

In B cell lymphoproliferative disease, ImmunoGlobulin Heavy chain (IGH) gene rearrangements are powerful markers able to identify the variation patterns of the clonal subpopulations. The IGH rearrangement is a unique DNA sequence that is generated during physiological recombination event occurring in pre-B lymphocytes and further modified in the germinal center during somatic hypermutation process [3]. Indeed, deletions as well as random insertions of nucleotides among the VDJ gene segments of the IGH genes create a huge junctional diversity. Such a highly diverse junctional repertoire gives rise to unique *fingerprint-like* sequences that are different in each healthy B-lymphoid cell (*polyclonal*), but constant in tumour population (*monoclonal*) [4] that retains the IGH rearrangement of the B cell giving rise to the tumour clone.

Markers detection and Minimal Residual Disease (MRD) monitoring are currently part of the routine clinical management of patients affected by Acute Lymphoblastic Leukemia and currently under validation in other B-mature lymphoid tumours, as Mantle Cell Lymphoma (MCL) [5], Follicular Lymphoma [6] and Multiple Mieloma [7]. In this context, the term MRD monitoring is used to define any approach aimed to detect and quantify residual tumour cells beyond the sensitivity level of routine imaging and laboratory techniques. Basically, in many clinical trials MRD is monitored by Polymerase Chain Reaction (PCR) based methods with the aims to predict therapeutic responses and guide clinical decisions to minimize the likelihood of clinical relapse [8]. Several studies [9, 10] show that clonal IGH rearrangements detection and MRD monitoring based on these markers are powerful early predictors of therapy response and outcome in B-mature lymphoid tumours. Currently, Sanger sequencing and Allele-Specific Oligonucleotides quantitative-PCR (ASO q-PCR) are the best approach for these purposes and MRD monitoring techniques standardization has been obtained in the context of the international *Euro MRD* group.

Although ASO q-PCR is able to detect one clonal cell out of 500.000 analyzed cells (reaching a sensitivity of up to a dilution of 1^−05^) [4], it has a number of limitations including (i) failures in marker identification, especially in somatically hypermutated neoplasms or when the tumour tissue infiltration is low, (ii) technical complexity, especially in the design of patient-specific reagents based on the main clone found in diagnostic samples and (iii) false-negative results due to clonal evolution events [11].

In this context, Next-Generation Sequencing (NGS) technology might overcome the limitations of the standardized ASO q-PCR MRD method thanks to its theoretically higher feasibility and sensitivity. A good correlation of MRD results between the two techniques has been already shown in [11] (*p*-value <0.001, *R*=0.791), with excellent concordance in 79.6% of the analyzed cases.

Moreover, NGS MRD approach might provide a full repertoire analysis through multi-clones detection at diagnosis and it gives the opportunity to monitor all the neoplastic clones at several follow ups. However, this issue requires suitable computational algorithm. Actually, the large volume of data, collected thanks to the advent of deep sequencing technologies, raises multiple challenges in data storage and data analysis, to efficiently extract new knowledge from the biological processes under study.

In literature, there are several tools as JoinSolver [12], HighV-QUEST [13], iHMMune-align [14], SoDA2 [15],VDJSeq-Solver [16], ARRest/Interrogate [17] and ViDJil [18] currently implemented for marker screening and detection of IGH rearrangements on a set of reads obtained from deep sequencing experiment of a single sample. Details about all cited algorithms are reported in the Additional file 1.

In this paper we present a new tool called HashClone, an easy-to-use and reliable bioinformatics suite that provides B-cells clonality assessment and MRD monitoring *over time*. HashClone is composed of four C++ applications for the data processing and a HTML5+Javascript application for the data visualization. The HashClone strategy is composed of three steps: the first and second step implement an *alignment-free* prediction method that identifies a set of putative tumour clones belonging to the repertoire of the patient under study. In the third step the IGH variable region (IGHV), diversity region (IGHD) and joining region (IGHJ) identification is obtained by the alignment of rearrangements with respect to the ImMunoGeneTics information system (IMGT) reference database [19].

In this paper, we tested the performance of HashClone on data derived from MCL patients, in which IGH rearrangements were analyzed using NGS approach in order to assess the major B-cell clone in the diagnostic sample and to monitor the MRD. The results were also compared with data obtained by the standardized approach for MRD monitoring, the ASO q-PCR.

## Methods

The whole experimental and computational methodology presented in this paper is outlined in the Additional file 2: Figure S1. In the following details about wet lab procedures and the HashClone algorithm are reported.

***Patients and genomic DNA recovering*** Biological samples were collected from five patients affected by MCL enrolled in *Fondazione Italiana Linfomi* prospective clinical trial (EudraCT Number 2009-012807-25). Samples were recovered at diagnosis and for three out of five patients also during fixed time points planned by clinical trials. All of them provided written informed consent for the research use of the biological samples and all the procedures were conducted in accordance with the Declaration of Helsinki. See Additional file 1 for more details. Mononuclear cells were obtained using Ficoll density separation (Sigma-Aldricht; Germany) or blood lysis from peripheral blood or bone marrow samples; genomic DNA (gDNA) was extracted according to the manufacturer instructions (LifeTechnologies). The features of the samples analyzed are reported in Additional file 3: Table S1.

***IGH rearrangements screening and MRD monitoring*** IGH rearrangements screening and MRD study were performed using both an NGS approach and the gold standard techniques, i.e. Sanger sequencing and ASO q-PCR.

***Next generation sequencing approach*** The DNA libraries were prepared using 500 ng and 100 ng of gDNA by two-steps PCR approach: in the first round, the IGH regions were amplified using FR1 BIOMED II primers [20], modified with an universal Illumina adapter linker sequence; while in the second PCR round, Illumina specific indexes (Illumina; Sigma-Aldrich) were incorporated to the first round PCR IGH amplicons [21]. After a Bioanalyzer QC control (Agilent), the purified PCR products were serially dilute and pooled to a final concentration of 9pM adding 10% PhiX. The sequencing run was carried out by Illumina V2 kit chemistry 500 cycles PE on MiSeq platform. A polyclonal sample, called buffycoat DNA, and negative control (water or HELA cell line) were added to each run. More details are reported in the Additional file 1.

***Sanger sequencing and ASO q-PCR approach*** Diagnostic gDNA was screened for IGH rearrangements using consensus primers (Leader and Framework Regions (FR) 1 and 2), as previously described [22]. Purified post PCR products were directly sequenced and analyzed using the IGH reference database published in IMGT/V-QUEST tool (http://imgt.org) [23]. MRD monitoring was conducted by ASO q-PCR on 500 ng of gDNA, using patient specific primers and consensus probes designed on Complementarity-Determining Region 2 (CDR2) sequences, on CDR3 and FR3 IGH regions, respectively [24]. MRD results were interpreted according to the ESLHO-Euro MRD guidelines [4].

### The HashClone algorithm

The HashClone strategy is organized on three steps. The *significant k-mer identification* (Step 1) and the *Generation of read signatures* (Step 2) implement an *alignment-free* prediction method that identifies a set of putative tumour clones from patient’s samples; while in *Characterization and evaluation of the cancer clones* (Step 3) the IGHV, IGHJ and IGHD identification is obtained via the alignment of rearrangements with respect to the IMGT reference database [19]. A detailed description of these three steps is now reported.

#### HashClone - description of the strategy

**Significant k-mer identification (Step 1).** In this step the entire set of reads for each of the *n* patient’s samples is scanned and a set of sub-strings of length *k*, namely k-mers, is generated using a *sliding window* approach. For instance given the read *ATCCCGTC* the following k-mers with *k*=3 are generated: ATC, TCC, CCC, CCG, CGT and GTC.

Formally, given an alphabet $\mathcal {L}=\{A,C,T,G\}$ where the letters correspond with DNA-bases we define *ρ*, namely *read*, as a string over $\mathcal {L}$ of arbitrary length *m*, and $A^{*}_{k}$ as the set of strings of length *k* constructed from $\mathcal {L}$. Then, $A^{\rho }_{k} =\left \{\alpha _{1}^{k},\alpha _{2}^{k+1},\ldots,\alpha _{m-k+1}^{m}\right \}$ is the set of strings of length *k* generated from *ρ* using *sliding window* approach s.t. $\alpha _{p}^{k+p-1}$ is the sub-string of *ρ* starting at position *p*, spanning *k* characters and ending at *k*+*p*−1. We define the function: 
1


s.t. for each k-mer returns a vector listing the total number of times this k-mer appears in any patient’s sample (i.e. k-mer frequencies for patient’s samples). Thus, $\mathcal {C}(\alpha)[i] = h$ with 1≤*i*≤*n*, iff k-mer *α* is present in *h* reads of the sample *i*.

Then, a k-mer *α* is defined as *significant* iff ∃1≤*i*,*j*≤*n* such that:

2 $$ \left\{ \begin{array}{rl} | log_{10}(\mathcal{C}(\alpha)[i])-log_{10}(\mathcal{C}(\alpha)[j])|\geq \tau, & if \; \mathcal{C}(\alpha)[\!i]\neq 0 \wedge \mathcal{C}(\alpha)[j] \neq 0 \\ log_{10}(\mathcal{C}(\alpha)[j])\geq \tau, & if\; \mathcal{C}(\alpha)[\!i]=0 \wedge \mathcal{C}(\alpha)[j]\neq 0 \\ log_{10}(\mathcal{C}(\alpha)[i])\geq \tau, & if\; \mathcal{C}(\alpha)[\!i]\neq 0 \wedge \mathcal{C}(\alpha)[j]=0 \\ \end{array} \right.  $$

where *τ* is a user-defined parameter. The choice of an appropriated *τ* value can impact on the capability of HashClone to identify clones. A detailed analysis about this aspect and the set of *τ* value used in the *Pilot1* and *Pilot2* experiments are reported in the Additional file 1.

Moreover, we introduce the following function:

3 $$ \mathcal{CH}: A^{*}_{k} \rightarrow \{\mathbf{TRUE,FALSE}\}  $$

that takes as input a k-mer *α* and returns ***TRUE*** iff *α* is a *significant k-mer* otherwise ***FALSE***. For instance, assuming *n*=3, *τ*=1, and $\mathcal {C}(ATC)=\langle 1000,2000,25000 \rangle $ then $\mathcal {CH}(ATC)$ returns ***TRUE*** because $|log_{10}(\mathcal {C}(ATC)[1])$ ${-log_{10}(\mathcal {C}(ATC)[3])|\geq 1}$.

Thus, $\mathcal {CH}$ function is used to derive the set of *significant k-mers* *Ψ*={*ψ*_1_,…,*ψ*_*t*_}.

**Generation of read signatures (Step 2).** This step takes as input the set *Ψ* of all the *significant k-mers*, and it generates the read signatures. Given a patient’s sample *i*, for each read *ρ* all its k-mers are analyzed to derive the corresponding read signature. A k-mer $\alpha \in A_{k}^{\rho }$ is selected iff *α*∈*Ψ*, then all the selected k-mers are combined to generate a read signature according to their positions in *ρ*.

For instance, considering the read *ATCCCGTC* and assuming CCC, CCG, CGT the only *significant k-mers* in the read the corresponding signature is CCGT. Defined *Γ*_*i*_={*γ*_1_,…,*γ*_*e*_} the set of read signatures obtained for the sample *i*, the function: 
4


returns the total number of reads of sample *i* in which the signature *γ* appears (i.e. signature frequency in patient’s sample *i*).

When the entire set of reads of sample *i* is scanned, the set of generated signatures *Γ*_*i*_ is processed to identify those similar (with respect to a fixed number of mismatches, insertions and deletions) using a Smith-Waterman algorithm. Practically in this correction step two signatures *γ*,*γ* ^′^∈*Γ*_*i*_ are considered similar if their alignment score computed by Smith-Waterman algorithm is greater than a specified threshold *T*. Hence, the signature *γ* with lower frequency is removed from the set of signatures and its frequency is added to the frequency of the other signature *γ* ^′^, i.e. $\mathcal {CS}(\gamma ')=\mathcal {CS}(\gamma)+\mathcal {CS}(\gamma ')$

**Characterization and evaluation of the cancer clones (Step 3).** This step takes as input the sets of signatures *Γ*_1_,…,*Γ*_*n*_ generated from each patient’s sample in the Step 2. We define the set of putative cancer clones *Δ* (initially empty), and the function: 
5


that for each clone *δ* returns a vector listing the total number of times this clone appears in any patient’s sample.

*Δ* is incrementally updated processing the signatures into each set *Γ*_*i*_ (starting from *Γ*_1_ to *Γ*_*n*_). For each signature *γ*∈*Γ*_*i*_ a similar putative cancer clone is searched in *Δ*. The similarity between a clone and a signature is evaluated using the same strategy proposed for the correction step. If a similar clone is not found then a new one identified by the signature sequence *γ* is inserted in *Δ* and its associated frequencies are defined as follows: let *γ* be a signature in *Γ*_*i*_ and *δ* the corresponding new clone then $\forall 1 \leq j \leq n \wedge j\neq i \Rightarrow \mathcal {CC}(\delta)[j]=0$, while for $j=i \Rightarrow \mathcal {CC}(\delta)[j]=\mathcal {CS}(\gamma)$. Instead, if a similar clone is found then its frequencies are updated as follows: let *γ* be a signature in *Γ*_*i*_ and the *δ* the corresponding similar clone then $\mathcal {CC}(\delta)[i]=\mathcal {CC}(\delta)[i]+\mathcal {CS}(\gamma)$.

Finally, the putative cancer clones in *Δ* are verified exploiting biological knowledge. Indeed, all the identified putative clones are analyzed and evaluated using IMGT reference database (http://www.imgt.org/download/GENE-DB/). For each clone, its best alignments with respect to V-GENE, J-GENE, and D-GENE are reported and ranked according to a similarity measure (i.e. matched bases divided matched and unmatched bases).

#### HashClone - implementation details

HashClone strategy described above, has been implemented thanks to tool suite specifically developed for this purpose. This tool suite, called HashClone, is composed of four C++ applications for data processing and one HTML5+Javascript application for the data visualization. Moreover, a Java-GUI has been also developed to simplify the data processing phase.

**Data processing** applications are:

- *HashCheckerFreq* takes as input reads of a patient’s sample and returns the corresponding set of k-mers associated with their frequency in the input reads. The k-mers and their frequency are stored in RAM as an associative array achieved through a C++ hash table class specifically implemented to optimize the trade-off between the memory utilization and the execution time. Observe that this class implements a *separate chaining* as collision resolution policy to deal with the case of different k-mers having a similar hash value.
- *CompCheckerKmer* takes as input all the k-mers derived by all the patient’s samples and their frequencies, and it analyses the k-mer frequencies in each patient’s sample to derive the set *Ψ* of *significant k-mers* (as defined in Eq. 2). This is achieved by exploiting an associative array, implemented through a *red-black tree* data structure. Hence, in this associative array the array keys are the k-mer sequences and the array values the k-mer frequencies. In this application, a *red-black tree* data structure was used (instead of hash table) because we are going to investigate the possibility of implementing an efficient correction step (up to *m* mismatches) based on the characteristic of this data structure.
- *HashCheckerSignature* takes as input the *significant k-mers* and the set of reads of *i* ^*th*^ sample and returns the set of read signatures for this sample (i.e. *Γ*_*i*_) with their frequencies. The k-mers are stored using the implemented hash table class, while the generated signatures are stored using red-black tree. A correction step identifying similar signatures (with respect to mismatches, insertions and deletions) is performed exploiting the implementation of the Smith-Waterman algorithm provided by SIMD Smith-Waterman C++ library [25]. In our implementation the *T* threshold previously introduced (in the Step 2, Generation of the read signature) to discriminate between similar reads is automatically derived as follows:
**IF** $max \left (size_{\gamma _{1}},size_{\gamma _{2}}\right) * 0.7>min \left (size_{\gamma _{1}},size_{\gamma _{2}}\right) $
**THEN** RETURN ($max (size_{\gamma _{1}},size_{\gamma _{2}}) *M $)
**ELSE** RETURN $((M *4/5 \!- \!MM*\!2/50\,-\,IN*\!2/10) * max (size_{\gamma _{1}},size_{\gamma _{2}}))$
  where $size_{\gamma _{1}}$ and $size_{\gamma _{2}}$ are the lengths of the two input signatures *γ*_1_,*γ*_2_, and M, MM and IN are the match, mismatch and insertion/deletion scores defined in the Smith-Waterman algorithm. Moreover, in our experiment we set M and MM score values equal to 2, and IN score value equals to 3. Observe that if the length of the smaller read is less than 70% of the length of the other then the reads *γ*_1_,*γ*_2_ are always considered different.
- *CompCheckerRead* takes as input the sets of signatures for each patient’s sample (i.e. *Γ*_1_,…,*Γ*_*n*_), and it derives the set of putative cancer clone *Δ*. Similar signatures among the samples are identified using the Smith-Waterman algorithm provided by SIMD Smith-Waterman C++ library. Then each identified putative tumour clone is analyzed to identify its best alignment with respect to V-GENE, J-GENE, and D-GENE. This task is performed thanks to a specifically developed aligner which uses a modified version of Smith-Waterman algorithm to find the best alignment of such clones with respect to the IMGT reference database.

**Fig. 1:**
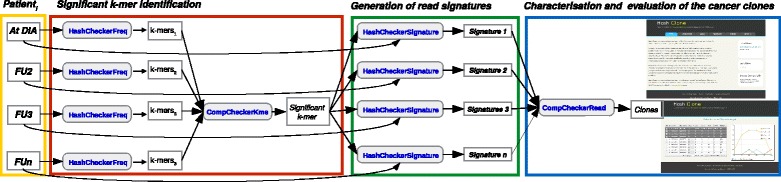
HashClone pipeline. The three steps at the basis of HashClone strategy are highlighted: the first step (red box) regards the *significant k-mer identification* considering all samples to be analyzed and generating the set of k-mers; the second step (green box) is focused on the *generation of read signatures* leading to the identification of the set of putative clones from patient’s samples; the third step (blue box) is dedicated to the *characterization and evaluation of the cancer clones*

Figure 1 shows how the above described C++ applications are combined in a workflow to implement HashClone strategy for B-cells clonality assessment and MRD monitoring from collected samples of a single patient. Practically, *HashCheckerFreq* is executed on each patient’s sample at a time to derive the k-mers and their associated frequencies. The collected set of k-mers generated by all the patient’s samples are the input of *CompCheckerKmer*, which computes the set of *significant k-mers*. Then, *HashCheckerSignature* is run on each patient’s sample to obtain the set of read signatures from the set of *significant k-mers*. Finally, *CompCheckerRead* is executed to derive the putative clones from the read signatures obtained by all patient’s samples. It is worth noting that since *HashCheckerFreq* and *HashCheckerSignature* are called on each patient’s sample then they are independent tasks and can be performed in parallel. Moreover, a Java GUI is provided to simplify the execution of this workflow. The tool suite and its associated Java GUI can be downloaded at the following address http://tanto.unito.it/WebVisual/.

**Data visualization** The developed application is a web application (http:/tanto.unito.it/WebVisual/) based on *jQuery*, a cross-platform JavaScript library which provides capabilities to create plug-ins on top of the JavaScript library. The web application visualizes the cancer clones in a *data-grid*, in which the first column called *Signature* reports all the *significant k-mers* are combined to generate the read signatures used to define the set of putative cancer clones; the second column namely *Clone* reports a representative read for each read signature; the next six columns show the best IGHV, IGHD, and IGHJ alignments with their associated identity values, and the remaining columns report the clone frequency in each sample.

Exploiting the functionality provided by the *jqxGrid* widget, the user can easily manipulate and query the data presented in the data-grid. For instance all the clones can be ordered with respect to each column or set of columns, and they can be filtered according to their frequencies or the occurrence of a specific sub-sequence. Then, the frequencies of tumour clones can be plotted and graphically compared using *Flot*, a JavaScript plotting library for *jQuery*. The obtained graph can be also exported as a png file.

## Results

### Patient samples and study design

Five MCL patients (PatA-E) were investigated for IGH detection and MRD monitoring using a new designed amplicon-based NGS approach. Two Pilot studies, namely *Pilot1* and *Pilot2* were performed, details about the sample are summarized in Additional file 3: Table S1. In *Pilot1* the five diagnostic samples and two (for PatD) and three (for PatA, B, C, and E) artificial dilution samples were analyzed. These samples were prepared diluting the diagnostic material in a pooled DNA derived from healthy subjects (“buffycoat”); the same buffycoat was included in the experiment, as polyclonal control. The 19 libraries were prepared using 500 ng of gDNA and sequenced as described in Material and Methods section. The data are available at http:/tanto.unito.it/WebVisual/. The average number of reads in each sample is equal to 481,289 (range: from 328,950 to 1,042,206 reads). The buffycoat sample contains 301,772 reads and the negative control (water) contains 466,348 reads. The quality check of the runs was performed using FastQC software (http://www.bioinformatics.babraham.ac.uk/projects/fastqc/) among the features considered the base quality (average value equals to 36) and the N content passed the check.

In *Pilot2* the five diagnostic samples and three (PatA) or four (PatB and E) real FU samples were sequenced. To test the efficiency of our wet lab procedures, 14 libraries were prepared reducing the gDNA input to 100 ng each. The average number of reads is equal to 316,789 (range: from 6,554 to 1,509,538 reads), while the buffycoat sample contains 478 reads and the negative sample (HELA cell line not carrying IGH rearrangements) contains 788 reads. As performed in *Pilot1*, we checked the quality of the data by FastQC software, but both base sequence quality (average value equals to 20) and N content features failed the check.

### Strategy for B-cell clones selection and biological validation

Five and three runs of HashClone were executed, one for each patient of *Pilot1* and *Pilot2*, respectively. Each run simultaneously analyzed the diagnostic sample and all artificial or clinical follow ups; the command lines used are reported in the Additional file 1. HashClone output displays the entire list of the identified B-cell clones associated with the frequency value, the IGH rearrangement (in terms of VDJ genes and alleles), and homology identity values. Among all the reported B-cell clones, it is necessary to define the predominant clones that should be followed for MRD purpose. For this reason, we designed a *filtered strategy* composed of two phases.

In the *Phase-A* we selected a set of predominant clones based on the frequency values observed in the diagnostic samples. As reported by Faham and colleagues in [26] any clonotype associated with low frequency value was prudentially not considered representative of the disease. The authors indicated a threshold of 5% that, in our experiments corresponds to 100 reads. Thus only the clones associated with a frequency value major than 100 in the diagnostic sample were considered.

In the *Phase-B* we considered the identity values associated with each B-cell clones: only the clones associated with more than 80% of homology in each IGHV, IGHD, and IGHJ genes are considered.

### Clonality and major B-cell clone detection

**Clonality.** The set of B-cell clones obtained by HashClone on both the *Pilot1* and *Pilot2* are processed following the *filtered strategy* presented above. In the diagnostic samples of the five patients of *Pilot1*, HashClone identified an average value of 1547 clonotypes (min 870, PatD; max 2149, PatC). The application of the *Phase-A* selected on average 38 clones of which on average 22 B-cell clones were retained in the analysis after the *Phase-B*. The average number of reads supporting these selected clonotypes is 100,929.

In *Pilot2* HashClone identifies an average value of 96 clonotypes (min 77, PatE; max 278, PatB). The *Phase-A* filters out around 18% of the clonotypes: on average 18 clones were passed to the *Phase-B*. On average 6 clones passed the *Phase-B*, the average number of reads supporting the selected clonotype is 141,570. Details about the results in both the Pilot studies are reported in Table 1.

**Table 1:** Clonotypes identified with HashClone analysis and IMGT validation

			Phase A	Phase B
Study	Patient	Clonotype	Clonotype with	Clonotype with
	(only diagnosis samples)	identified	frequency >100	VDJ homology >80%
*Pilot 1*	A	1616	12	7
	B	1703	59	33
	C	2149	72	44
	D	870	10	5
	E	1398	35	21
	**Average value**	**1547**	**38**	**22**
*Pilot 2*	A	96	18	6
	B	278	72	11
	E	77	5	0
	**Average value**	**150**	**32**	**6**

For each patient of both Pilot studies the total number of identified clonotypes (third column) is reported. The number of clonotypes with a frequency greater than 100 were selected and passed the Phase A are reported in fourth column. Then from the Phase A, clonotypes with a VDJ homology greater than 80% were selected and passed the Phase B (fifth column). The average value are reported in bold

**Fig. 2:**
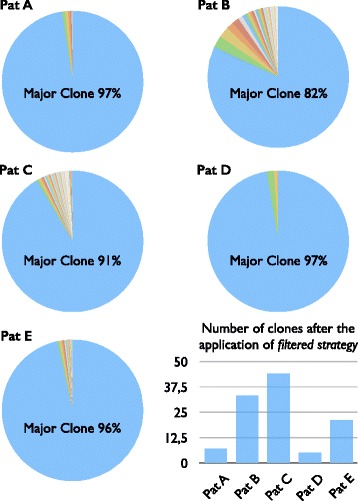
Clonality analysis in MCL patients. Pie plots showing the distribution of the frequency percentage associated with the B-cell clones passed the *filter strategy* in the five diagnostic samples of *Pilot1*. Into each pie plots it is reported the frequency percentages associated with the major clone. The histogram reports the number of B-cell clones passed the *filter strategy* in each patient

In *Pilot1* each of the five diagnostic samples clearly displayed one major clone with an average frequency of 93% (min 82%, PatB; max 98% PatA); while the other identified B-cell rearrangements showed an average frequency value equals to 7% (min 2% PatA; max 18% PatB), see Fig. 2 and Additional file 4: Figure S2. In *Pilot2* the predominant clone is easily identified since its average frequency is 88% (min 73%, PatB; max 99% PatE) while the other B-cell clones showed an average frequency value of 12%. See Additional file 4: Figure S2 for more details.

**Major B-cell clone detection.** Before dealing with the details about the HashClone results accuracy, we tested the performance of the IGH alignment implemented in HashClone (i.e. Step 3) using the Stanford_S22 dataset. We considered the paper of Jackson et al. [27] in which the authors evaluated the performance of seven algorithms handling the thousands of IGH rearrangements in Stanford_S22 dataset to identify the IGHV, IGHD and IGHJ assignments and compare these back to the known genes from the inferred genotype for the subject. The overall error for HashClone is equal to 1.8% that is the lowest value compared to the overall error percentages reported by Jackson, ranging between 7.1% (using iHMMune-align algorithm) and 13.7% (using Ab-origin algorithm).

For each patient the predominant clone identified by HashClone was compared with the IGH monoclonal rearrangement identified by Sanger sequencing, in terms of IGHV, IGHD and IGHJ nucleotide homology, using BLASTn algorithm http://blast.ncbi.nlm.nih.gov. Four out of five diagnostic samples of *Pilot1* (PatA, C, D and E) showed exactly the same IGH rearrangement, in terms of IGH gene annotation and 100% nucleotide homology with respect to the Sanger sequence. Also Patient B showed the same rearrangement excepted for three nucleotide mismatches. On the other hand, a lower nucleotide homology (ranging from 44 to 66%) was noticed in *Pilot2*, due to the high number of unknown base calls (N) introduced by sequencing in the variable regions. Nevertheless, HashClone was still be able to assign the correct IGHV and IGHJ annotations, perfectly comparable with the Sanger results. These results are reported in Table 2.

**Table 2:** HashClone and Sanger Sequence comparison

	

The label of the table should be changed with the following sentence: This table reports the comparison in terms of IGHV, IGHD, and IGHJ nucleotide homology between the predominant clone identified by HashClone and the IGH monoclonal rearrangement identified by Sanger sequencing for each patient. Last column reports the homology between the two sequences as difference in nucleotide content and percentage. Bold and underline sequences correspond to the patient specific insertions among IGHV, IGHD, and IGHJ rearrangement. Red nucleotides in the sequences are those who differ between two sequences. N: unknown base calls

### Minimal residual disease monitoring

To monitor the MRD, HashClone tracks the clonotypes evolutions analyzing simultaneously the data from the diagnostic and the serial dilutions (*Pilot1*) or FU samples (*Pilot2*). Therefore, we compared the HashClone performance with the standardized results of the classical ASO q-PCR.

To make the MRD quantifications comparable between the two approaches, we set up a proportion between the total reads number of the major MCL clone at diagnosis (HashClone) and the ASO q-PCR value. In details, patients A, C, D, and E had a high tumour infiltration (ASO q-PCR value of 1E+00 according to EuroMRD guidelines) [4]; while patient B started from an ASO q-PCR value of 1*E*−01, according to a lower tumour infiltration. These data are confirmed by a 2.5% *C* *D*5^+^/*C* *D*19^+^ MCL cells rate by flow cytometry.

**Fig. 3:**
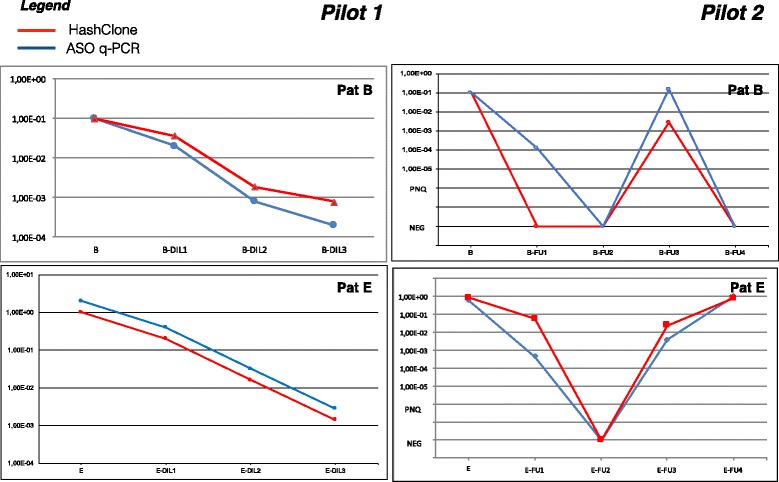
MRD trend comparison. MRD trend obtained from ASO q-PCR (blue line) and HashClone (red line) of Patient B and E of *Pilot1* and patient A and E of *Pilot2*

**Fig. 4:**
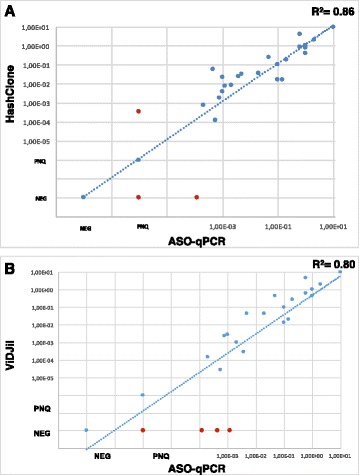
Correlation analysis. Scatter plot of the correlation analysis between HashClone and the ASO q-PCR data (Panel **a**) and between ViDJil and the ASO q-PCR data (Panel **b**). In Panel **a**, three discordances (red dots) are detected, one of them is quantifiable only by HashClone. While in Panel **b** there are four samples quantifiable only by ASO q-PCR. NEG, Negative; PNQ, Positive Not Quantifiable

HashClone was able to perfectly extract the MRD trend kinetics in the dilution/FU samples of the five MCL patients in both Pilot studies. Figure 3 reports the trends of PatB and Pat E (*Pilot1*) and PatA and PatE (*Pilot2*). Overall, the correlation analysis showed a high concordance between ASO q-PCR and the NGS technology (*R* ^2^=0.86), see Fig. 4 Panel a. Indeed 30 out of 33 points are concordant: in *Pilot1* HashClone overestimates the frequency value in one case point; in *Pilot2* ASO q-PCR overestimates the frequency value in two cases.

### Evaluation of Hashclone accuracy with respect to ViDJil algorithm

We compared the accuracy of HashClone with respect to ViDJil algorithm. At the best of our knowledge, ViDJil is the only tool currently able to analyze the high-throughput sequencing data from lymphocytes, to extract IGHV, IGHD, and IGHJ junctions and to gather them into **clones** for quantification. ViDJil quantifies the clonotype abundances through a first ultrafast prediction of putative rearrangements by a seed-based heuristic analysis and it outputs a window overlapping the CDR3 with the IMGT reference database. The putative clone sequence identified is further processed to obtain its full IGHV, IGHD, and IGHJ segmentation. Moreover, ViDJil can carry out the MRD analysis thanks to a web multi-sample application able to track selected clones in the diagnostic samples through different runs on different FU samples.

The strategy used to analyze the ViDJil results is composed of two phases: the *Phase-A* is the same implemented for HashClone, in the *Phase-B* since ViDJil associates the clones with the VDJ genes and alleles without reporting the homology values, we consider only the clones associated with one IGH rearrangement.

The set of B-cell clones obtained by ViDJil on both the *Pilot1* and *Pilot2* and those filtered by *Phase-A* and *Phase-B* are reported in Additional file 5: Figure S3. More details about the number of reads associated with each clone are reported in Additional file 6: Figure S4. In *Pilot1* ViDJil is able to detect the major B-cell clone in all patients, the CDR3 regions detected in patients A, C, D and E have 100% homology with respect to the Sanger sequence, while patient B has an homology value equal to 93%, as reveled by HashClone. In *Pilot2* the elevated number of N base calls masking the CDR3 regions did not allow ViDJil to correctly annotate the IGHV, IGHD, and IGHJ in any patient, so that the nucleotide homology value dropped to 0 with respect to the Sanger sequence, see Additional file 7: Figure S5. In contrast, as described above, the HashClone performance was not hampered by the number of N base calls in the *Pilot2*.

We also compared the MRD quantification of all samples of both *Pilot1* and *Pilot2* between ViDJil and the ASO q-PCR data. Figure 4 reports the correlation analysis of all samples between HashClone and the ASO q-PCR data (Panel a) and between ViDJil and the ASO q-PCR data (Panel b). It is worthwhile to note that the concordance between HashClone and ASO q-PCR is higher than the concordance between ViDJil and ASO q-PCR, 86% versus 80% respectively.

## Discussion

In this paper we have presented a new tool suite called HashClone. HashClone is an easy-to-use and reliable bioinformatics suite that provides B-cells clonality assessment and IGH-based MRD monitoring over time. To test its performances we analyzed two NGS experiments targeting the IGH rearrangements in samples obtained from patients affected by MCL.

Our results showed that HashClone was able to detect the major B-cell clone in MCL patients, these clonotypes are indeed confirmed through the classical Sanger sequencing approach. Moreover, HashClone efficiently analyzed NGS data to monitor the MRD, providing highly comparable data with respect to the standardized ASO q-PCR.

The HashClone strategy to identify a set of putative clones is composed of three steps: the first two steps implement an *alignment-free* prediction method that identifies the set of putative clones belonging to the repertoire of the patient under study. The advantage of using an *alignment-free* prediction with respect to alignment prediction methods (based on a reference genome) is twofold: (i) it may provide new rearrangements because no reference is used to select the putative clones, (ii) it may be more robust to detect genome-scale events as rearrangements, recombination, and duplications [28]. Moreover, the *alignment-free* prediction method provides an elevate accuracy, because the putative clones are identified through an *integrated* analysis of all the patient’s samples collected over time. Finally, the last step is focused on the identification of the germline origins of IGH rearrangements based on alignment of the putative B-cell clones with respect to the IMGT reference database [19]. Notice that the current tool implementation allows the users to exploit different datasets since the database is not embedded in the code leading to broadly applications of HashClone to biological projects dedicated to the clonality detection from NGS data.

To assess the accuracy of HashClone to identify the major B-cell clone and to monitor the MRD we compared its performance with respect to the results obtained by ViDJil tool. Indeed, at the best of our knowledge, ViDJil is currently the only available tool able to analyze the high-throughput sequencing data from lymphocytes, to extract VDJ junctions and to gather them into **clones** for quantification.

The comparison was done on two MCL pilot studies generated using either 500 ng (*Pilot1*) or 100 ng (*Pilot2*) of gDNA as input in library preparation.

The two experimental protocols considered reflect different clinical/biological situations. *Pilot1* reproduces in the NGS setting the optimal requirements of a classical IGH screening experiment and a dilution curve. On the other hand *Pilot2* investigates the effects of a decrease in DNA quantity, mimicking a *real-life* situation that typically occurs in the routine of haematological laboratories. The restricted DNA availability can be due to the low cellularity of the biological samples (i.e low disease infiltration or material lack) or to specific sample conditions (i.e DNA extracted from formalin fixed paraffin embedded-FFPE- samples, or cell-free DNA from serum, plasma, or urine).

Our NGS experiments showed that, even though the mean number of reads obtained from the two studies was similar (481,298 *Pilot1* and 316,789 *Pilot2*), the base sequence quality was poorer in the *Pilot2*. This is reported by the base N content (FastQC check failed for the *Pilot2*) and the base sequence quality (mean value of 36 in *Pilot1* compared to a mean value of 20 in *Pilot2*). The limited quality of the *Pilot2* data is reflected on a very low homology level of the CDR3 regions with respect to the Sanger sequence (average value of 99% in *Pilot1* with respect to an average value of 58% in *Pilot2*, *p*-value=0.02, computed by Student’s t-test). HashClone and ViDJil correctly identified the major clones in *Pilot1*. However, in *Pilot2* the elevate number of N base calls masked the IGHD region and reduced the nucleotide homology, leading to a decrement in the efficiency of ViDJil. In contrast, HashClone was able to identify the major clone in all the diagnostic samples. Moreover, in MRD monitoring we computed the concordance between the results obtained from the algorithms with respect to the ASO q-PCR data. Also in this analysis the performance of HashClone outperformed the ViDJil results (concordance percentage: 86% HashClone, 80% ViDJil).

Actually, Hashclone has two main distinct features with respect to VIDJil, the first is the *reference free* strategy, that allows Hashclone not to use biological knowledge until the last step in which it is necessary to assign to the putative clonotype the IGHV, IGHD and IGHJ composition. Secondly, Hashclone is specifically designed for the MRD detection working simultaneously on all sets of samples belonging to a patient. Instead, VIDJil is not specifically designed to simultaneously work on all samples. Indeed, VIDJil provides a set of additional tools able to fuse the set of rearrangements obtained from each sample generating the set of clone associated with the temporal trend.

Future analysis on the clones obtained by HashClone will be implemented using statistical methodologies [29, 30]

## Conclusions

HashClone tool can efficiently support the researchers in the identification of B-cell clonality in NGS-based experiments and in the monitoring of MRD in lymphoproliferative disorders. Indeed, the reported results on the considered two experimental settings demonstrated the HashClone ability to correctly detect the major B-cell clones and to precisely follow them in several samples even when the nucleotide sequences are characterized by the inclusion of substantial proportions uncertain nucleotide assignment. Moreover, the provided GUI for HashClone should improve the tool usability and facilitate the clonality visualization over time.

## Additional files

Additional file 1 Experimental and Computational details. Additional materials in which it is deeply described: the pilot studies, the sample processing and genomic DNA extraction, the Minimal Residual Disease analysis using classic PCR approach, the Minimal Residual Disease analysis using IGH amplicon based on deep sequencing approach, the State of the art of algorithms for IGH analysis, the Pilot study analysis by HashClone, and HashClone performance on IGH alignment using StandfordS_22. (PDF 323 kb)

Additional file 2 **Figure S1.** Whole methodology. The whole experimental and computational methodology presented in this paper. (PDF 885 kb)

Additional file 3 **Table S1.** Experimental details. The main features of the samples analyzed. ^∗^ these samples were analyzed in both *Pilot1* and *Pilot2*. (PDF 29.7 kb)

Additional file 4 **Figure S2.** Clonotypes quantification by Hashclone. Hashclone identifies an average number of clones equals to 21 in *Pilot1* and 32 in *Pilot2*. In the last column of the table is reported for each major clone the number of reads associates to it with respect to the total number of reads. The same data are also reported for the other clones identified. (PDF 52.7 kb)

Additional file 5 **Figure S3.** Clonotypes identification by ViDJil. The clonotypes identified by ViDJil in *Pilot1* and *Pilot2* are reported in the third column. In the fourth column are reported the clones passed the *Phase A* while in the fifth column there are the number of clones passed the *Phase B*. (PDF 46.4 kb)

Additional file 6 **Figure S4.** Clonotypes quantification by ViDJil. ViDJil identifies an average number of clones equals to 37 in *Pilot1* while in *Pilot2* it does not identified any clonotypes. In the last column of the table is reported for each major clone the number of reads associates to it with respect to the total number of reads. The same data are also reported for the other clones identified. (PDF 51.9 kb)

Additional file 7 **Figure S5.** ViDJil and Sanger Sequence comparison. Nucleotide alignments between the complementary region 3 sequences (CDR3, indicated in bold and underline) Sanger sequence and the sequence identified by ViDJil. (PDF 60.9 kb)

## Abbreviations

ASO q-PCR: Allele-Specific Oligonucleotides quantitative-PCR
BM: Bone Marrow
CDR3: Third Complementarity-determining region
FU: Follow Up
gDNA: Genomic DNA
IGH: ImmunoGlobulin heavy chain
IMGT: ImMunoGeneTics information system
MCL: Mantle cell lymphoma
MRD: Minimal residual disease
NGS: Next generation sequencing
PB: Peripheral blood PCR: Polymerase chain reaction
