# Supplementary material for: HashClone: a new tool to quantify the minimal residual disease in B-cell lymphoma from deep sequencing data
Source: BMC Bioinformatics. 2017 Nov 23;18:516. doi: 10.1186/s12859-017-1923-2 (PMC5701356; doi:10.1186/s12859-017-1923-2)
Supplement: Supplementary file 1 — Experimental and Computational details. Additional materials in which it is deeply described: the pilot studies, the sample processing and genomic DNA extraction, the Minimal Residual Disease analysis using classic PCR approach, the Minimal Residual Disease analysis using IGH amplicon based on deep sequencing approach, the State of the art of algorithms for IGH analysis, the Pilot study analysis by HashClone, and HashClone performance on IGH alignment using StandfordS_22. (PDF 323 kb) [file 12859_2017_1923_MOESM1_ESM.pdf]

1 Patients

In total, five patients affected by mantle cell lymphoma (MCL) and enrolled in Fondazione Italiana Linfomi (FIL) MCL0208 prospective clinical trial (EudraCT Number 2009012807-25) were investigated.
In *Pilot1*, for each patient, samples were prepared using a 10-fold dilution of the diagnostic tissue in pooled DNA recovered from five healthy subjects, see Figure 1 Panel A. The samples of the *Pilot2* were recovered at diagnosis (DIA, n=3) and during fixed time points during clinical trial course as follow: after induction treatment (n=3), post high dose consolidation therapy (n=2), post autologous stem cells transplant (ASCT) (n=2) and during observation after ASCT (n=4), details about the time schedule are reported in Figure 1 Panel B. All the patients provided written informed consent for the research use of the biological samples and all the procedures were conducted in accordance with the Declaration of Helsinki.

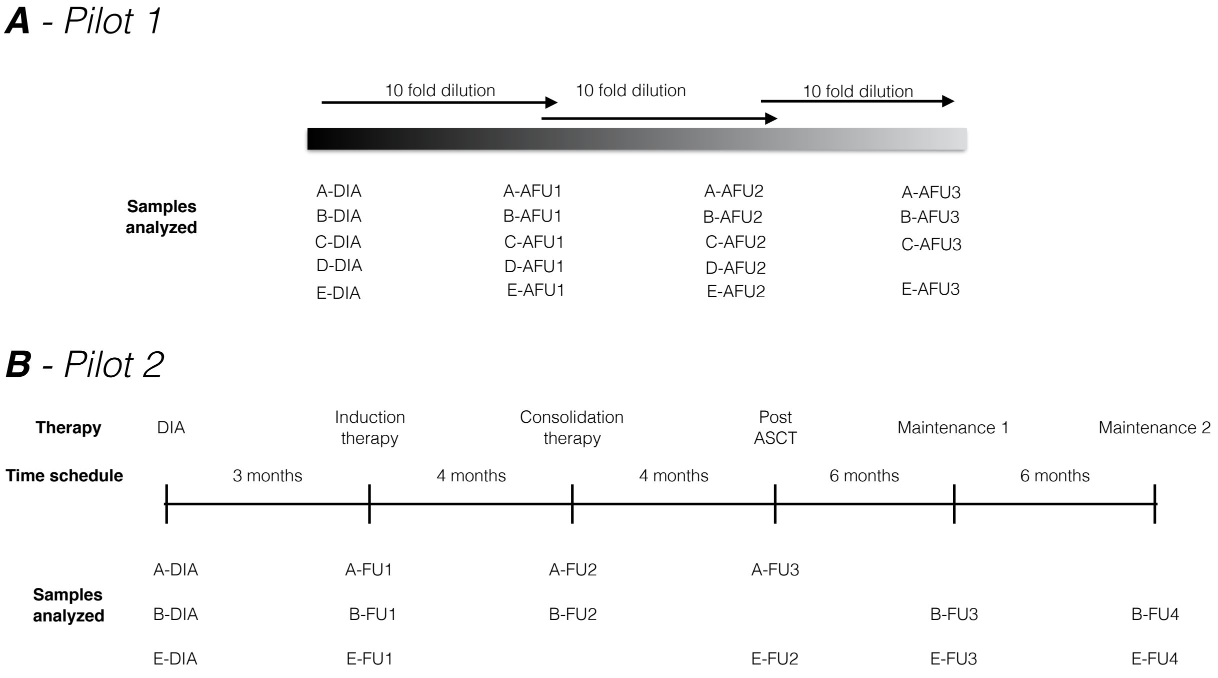

Figure 1: **A** Dilution gradient for samples of *Pilot1*. **B** The time line for the *Pilot2* patients .

2 Sample processing and genomic DNA extraction

For Diagnostic and follow up samples 5 ml of bone marrow (BM) and/or 10 ml of peripheral blood (PB) were collected in Sodium citrate or Lithium-heparine vacutainers. Cells recovery was performed using Ficoll density stratification and red blood cells lysis, obtaining mononuclear cells (MNCs) and total white blood cells (Total WBCs) for PB and BM respectively. Firstly, for MNCs isolations PB samples were diluted with NaCl 0,9% solution (ratio 1:2)

and then stratified on Ficoll layer (Sigma-Aldricht; Germany). After density separation, performed as recommended by manufacturer's instructions, MNCs were isolated and stored at -80°C in dried pellets until genomic DNA (gDNA) extraction. WBCs were recovered from bone marrow (BM) mixed to lysis buffer 1X [NH4Cl, KHCO3 and EDTA (pH7,3)] (Qiagen, Germany) (ratio 1:2). After incubation at room temperature for 10 minutes, the samples were centrifuged twice at 1500 rpm for 15 and 10 minutes, respectively. Total WBCs were stored as dry pellets at -80°C until gDNA extraction.

gDNA extraction was extracted from MNCs and total WBCs using DNAzol reagent (Thermo Scientific). Briefly, MNCs and WBCs were lysed in 1 ml of DNAzol and then centrifuged. gDNA precipitation and washes were performed with 100-70% ethanol solutions.

gDNA quantity (ng) and purity (OD ratio A260/A280 and A260/A230) were evaluated by Nanodrop2000 Spectrophotometer (Thermo Scientific). Housekeeping gene control amplification (p53 exon 8) [1] was performed on MNCs and total WBC samples in order to assess gDNA integrity.

3 Minimal Residual Disease analysis using classic PCR approach

Immunoglobulin heavy chain (IgH) rearrangements screening was performed for all diagnostic samples in order to detect a molecular marker able to track the disease level during clinical follow ups. Briefly, IGH rearrangements were detected by PCR using forward primers annealing to the VH-Leader (VH-L) or VH-Framework Regions (VH-FR) and a reverse primer complementary to JH region. For IGH marker screening 500 ng of gDNA were amplified as previously described [2].

PCR products were purified using QIAquick PCR Purification Kit (Qiagen, Germany), the purified samples were diluted to 10 nM and pooled at equimolar concentrations (21 and 16 pooled samples for *Pilot 1* and *Pilot 2*, respectively) and directly sequenced by Sanger approach. FASTA files were manually checked for base quality, discarding sequencing with high level of background signal. Then, Sanger results were aligned to B-cell IGH reference database using IMGT/V-QUEST tool (`http://imgt.org`) in order to detect IGHV, IGHD, and IGHJ rearrangements, IGH rearranged family, and nucleotide homology compared to germline sequence [3].

Based on IMGT analysis, Minimal Residual Disease (MRD) were carried out following allele specific oligo (ASO) primers approach. In detail, with this strategy reverse primer was designed on complementary regions (CDR) 3, where IGH rearrangement process introduces patient allele specific nucleotides, called N insertions. Forward primers and probes were designed on more conserved CDR2 and Framework Regions (FR) 3, respectively [4]. MRD was monitored by quantitative PCR, using 500 ng as input gDNA quantity, as previously described [5].

For each MCL patient, a 10-fold standard curve was prepared diluting diagnostic sample in gDNA pool from five healthy donors. The MRD quantification in follow ups samples was carried out, extrapolating data from standard curve and according to the standardized Euro MRD guidelines [6].

4 Minimal Residual Disease analysis using IGH amplicon based on deep sequencing approach

Each library (n= 19 *Pilot 1* and n=14 *Pilot 2*) was created as previously described [7] From 500 ng (Pilot 1) and 100 ng (Pilot 2) of gDNA a two steps PCR approach was used for the libraries preparation. In the first PCR round, a multiplex IGH amplification were performed by IGH FR1 Biomed-2 system (six forward primers annealing VH FR1 region and one reverse primer common for JH regions 2) [6], modified in 5' and 3' extremities with universal adaptors.

Oligo Name	Sequence 5'-3'
**univ-VH1-FR1**	gtaaaacgacggccagtGGCCTCAGTGAAGGTCTCCTGCAAG
**univ-VH2-FR1**	gtaaaacgacggccagtGTCTGGTCCTACGCTGGTGAAACCC
**univ-VH3-FR1**	gtaaaacgacggccagtCTGGGGGGTCCCTGAGACTCTCCTG
**univ-VH4-FR1**	gtaaaacgacggccagtCTTCGGAGACCCTGTCCCTCACCTG
**univ-VH5-FR1**	gtaaaacgacggccagtCGGGGAGTCTCTGAAGATCTCCTGT
**univ-VH6-FR1**	gtaaaacgacggccagtTCGCAGACCCTCTCACTCACCTGTG
**T7-JH-cons**	taatacgactcactatagggCTTACCTGAGGAGACGGTGACC

Figure 2: First round PCR-Biomed II primers

.

In the second PCR reaction, 1 ul of PCR products was amplified with tagged primers, composed by adaptors complementary and Illumina index sequences 3. The 2nd round PCR products were purified by Agencourt AMPure beads (Beckman Coulter) and quantified by Picogreen (Thermo Scientific). The purified samples were diluted to 10 nM and pooled at equimolar concentrations. Finally, a 9pM pooled library was sequenced on MiSeq platform (Illumina, San Francisco), with 500 cycles paired ends (PE) v2 chemistry obtaining a total coverage of about 190,000 reads for each sample analyzed. The read length was equal to 250 nucletides.

For sake of clarity, as previously published by Van Dongen JJM and co-workers [6], the protocol developed by BIOMED-2 Concern Action allows the detection of B clonality by PCR using a multiplex approach, that amplifies the entire IGH locus having a size of 100-360 bp [Watson CT Nature 2012]. Based on these evidences, in our experiments the libraries preparation was set following a two steps PCR approach, as described in the Material and Method section. In the first-round PCR, BIOMED II primers, annealing in IGH-FR1, amplified a region sized 310-350 bp. In the second PCR step, samples specific indexes of 80 bp length were incorporated to the at 5' and 3' sides of first-round PCR IGH amplicons in order to acquire sequences from multiple independently barcoded samples [8]. Overall, 400 bp libraries were sequenced with a v2-500 cycles PE, setting the Illumina instrument to trim the adapter sequences. After the sequencing, a preliminary analysis on forward and reverse reads showed a complete overlap between the reads, in particular for the CDR3 regions comprising the patient specific information used in the NGS-Sanger comparison. Based on these data, we analysed only the forward FASTQ files.

The *Pilot1* reveals a good quality in all fastq files in terms of quality sequence per base (mean value equals to 36, not value minor than 22) and in terms of the general nucleotide (N) content per base (mean value in all position equals to 1). Differently, in all samples of the *Pilot2* the quality per base has a mean value of 20 and the percentage of N at the 3' of the reads is more than 80% in each samples.

Oligo Name	Sequence
**III-D501-F**	AATGATACGGCGACCACCGAGATCTACACTATAGCCTACACTCTTTCCCTACACGACGCTCTTCCGATCTgtaaaacgacggccagt
**III-D502-F**	AATGATACGGCGACCACCGAGATCTACACATAGAGGCACACTCTTTCCCTACACGACGCTCTTCCGATCTgtaaaacgacggccagt
**III-D503-F**	AATGATACGGCGACCACCGAGATCTACACCCTATCCTACACTCTTTCCCTACACGACGCTCTTCCGATCTgtaaaacgacggccagt
**III-D504-F**	AATGATACGGCGACCACCGAGATCTACACGGCTCTGAACACTCTTTCCCTACACGACGCTCTTCCGATCTgtaaaacgacggccagt
**III-D505-F**	AATGATACGGCGACCACCGAGATCTACACAGGCGAAGACACTCTTTCCCTACACGACGCTCTTCCGATCTgtaaaacgacggccagt
**III-D506-F**	AATGATACGGCGACCACCGAGATCTACACTAATCTTAACACTCTTTCCCTACACGACGCTCTTCCGATCTgtaaaacgacggccagt
**III-D507-F**	AATGATACGGCGACCACCGAGATCTACACCAGGACGTACACTCTTTCCCTACACGACGCTCTTCCGATCTgtaaaacgacggccagt
**III-D508-F**	AATGATACGGCGACCACCGAGATCTACACGTACTGACACACTCTTTCCCTACACGACGCTCTTCCGATCTgtaaaacgacggccagt
**III-D701-R**	CAAGCAGAAGACGGCATACGAGATCGAGTAATGTGACTGGAGTTCAGACGTGTGCTCTTCCGATCTtaatacgactcactataggg
**III-D702-R**	CAAGCAGAAGACGGCATACGAGATTCTCCGGAGTGACTGGAGTTCAGACGTGTGCTCTTCCGATCTtaatacgactcactataggg
**III-D703-R**	CAAGCAGAAGACGGCATACGAGATAATGAGCGGTGACTGGAGTTCAGACGTGTGCTCTTCCGATCTtaatacgactcactataggg
**III-D704-R**	CAAGCAGAAGACGGCATACGAGATGGAATCTCGTGACTGGAGTTCAGACGTGTGCTCTTCCGATCTtaatacgactcactataggg
**III-D705-R**	CAAGCAGAAGACGGCATACGAGATTTCTGAATGTGACTGGAGTTCAGACGTGTGCTCTTCCGATCTtaatacgactcactataggg
**III-D706-R**	CAAGCAGAAGACGGCATACGAGATACGAATTCGTGACTGGAGTTCAGACGTGTGCTCTTCCGATCTtaatacgactcactataggg

Figure 3: Second round PCR-indexing primers. Adapater (uppercase nucleotides), index (red uppercase nucleotide), and universal linker sequences (lowercase nucleotides)

5 State of the art of algorithms for IGH analysis

In literature there are several tools for IGH analysis. At the best of our knowledge JOINSOLVER [9], IMGT HighV-QUEST [3], iHMMune-align [10], SoDA2 (Somatic Diversification Analysis 2) [11], VDJSeq-Solver [12], ARRest/Interrogate [13], and ViDJil [14] are implemented for marker screening and detection of rearrangements on a set of reads obtained from deep sequencing experiments. In the following, there is a brief description for each tool.

JOINSOLVER [9] is a web-based software program developed for human immunoglobulin recombination analysis created by the National Institutes of Health, National Institute of Arthritis and Musculosketelal and Skin Diseases and the Center for Information Technology. JOINSOLVER was developed to analyze the complementarity-determining region 3 (CDR3) of the immunoglobulin genes in human B cells which includes the IGHD gene and its junction with the IGHV and IGHJ genes. The length of the IGHD segment in CDR3 is extremely short (3-10 nucleotides) making the identification of this segment very difficult.

JOINSOLVER uses a consecutive matching approach to assign the IGHD segments and to limit the portion of the sequences that must be analyzed to identify IGH V - J regions. JOINSOLVER interrogates the input sequence to find the beginning of the CDR3 region that is characterized by conserved motif in most of the human IGHV germline genes. When, this segment is defined, JOINSOLVER screens the sequence in order to identify the limits of the IGHJ segment. Once the CDR3 region is identified, IGHV, IGHJ, and IGHD assignment is done comparing the sequences stored in IMGT reference database [15].

IMGT HighV-QUEST [3] is a web portal supported by the international ImMunoGeneTics information system (IMGT) consortium for the analysis of rearranged nucleotide sequences of the antigen receptors (immunoglobulins or antibodies and T cell receptors) obtained from deep sequencing data. IMGT HighV-QUEST is the high-throughput version of IMGT V-QUEST, it is able to analyze 500.000 nucleotide sequences per run. IMGT HighV-QUEST allows (i) the identification of the closest V, D and J genes and alleles, (ii) the IMGT/JunctionAnalysis, (iii) the description of mutations, and (iv) the characterization of IMGT clonotypes. The IMGT HighV-QUEST standard output includes a summary file which contains the IGHV, IGHD, and IGHJ annotation, the score of the alignment, the identity percentage and the identity nucleotides for each sequence. Moreover, the translated sequences, the junction frame and potential insertions/deletions among the rearrangement regions are reported. In addition to the summary file, IMGT HighV-QUEST reports more detailed files with the complete analysis of nucleotide sequences, aminoacids sequences, junction regions and possible nucleotide mutations.

iHMMune-align [10] is an alignment program that uses a Hidden Markov Model (HMM) to model the processes involved in human IGH gene rearrangement and maturation. iHMMune-align was developed by the School of Biotechnology and Biomolecular Sciences, School of Computer Science and Engineering, The University of New South Wales (Sydney, Australia). First of all, iHMMune-align identify the IGHV gene with a local alignment step of the sequence with the human IGHV germline repertoire stored in IMGT. The pre-alignment of IGHV gene allows iHMMune-align to calibrate the emission probabilities of the HMM through the estimation of somatic mutation amount over the sequence. Initially, the HMM is developed on rearranged sequences without mutations. The emission probabilities in the match states are re-computed to model the process of somatic mutation. This probability is adjusted to take into account the position of the putative mutations, the local sequence context and the effect of antigen selection. The HMM is finally aligned with the rearranged, mutated sequence, using the Viterbi algorithm. The program outputs the alignment corresponding to the optimal path along the HMM and reports the germline genes.

SoDA2 (Somatic Diversification Analysis 2) [11] is a tool based on a HMM that compute the posterior probabilities of candidate VDJ rearrangements of Ig genes and find those with the highest values among them. SoDA2 was developed by the Center for Computational Immunology, Computational Biology and Bioinformatics Program and the Department of Biostatistics and Bioinformatics (Durham,USA). SoDA2 aligns the target sequence with a consensus-like sequence of the V families to determine if the input sequence is a heavy, kappa or lambda chain. Then a pre-alignment step of V and J gene is performed in order to submit

to the HMM all V and J segments with highest likelihood alignments. The HMM is used to compute the emission probability in 10 states (see [11] for more details about the states). Then, the total probability of a proposed rearrangement is calculated using the forward and backward algorithms. The gene segments leading to the highest posterior probabilities are selected to identify the path with the highest posterior probability for each possible rearrangement through posterior Viterbi algorithm approach. The final output is composed of the top rearrangement candidate (highest posterior probability) and also rearrangements associated with high posterior probabilities in order to have a complete picture of the input sequences.

VDJSeq-Solver [12] is a tool for the identification of clonal lymphocyte populations from paired-end RNA Sequencing reads. The tool was developed by the Department of Control and Computer Engineering, Politecnico di Torino (Torino, Italy). The tool detects the main clone characterizing the tissue of interest by recognizing the most abundant V(D)J rearrangement in the sample. VDJSeq-Solver implement a unique pipeline composed of several existing tools: TopHat, Bowtie, BEDTools, BLAST alignment and Shrimp. VDJSeq-Solver performs a first alignment of the reads to the reference genome using both Bowtie and Tophat. The output of Bowtie contains the reads not mapped on the genome (VDJ unmapped); the output from Tophat contains all reads mapped on the reference genome that become the input of BEDTools to extract all reads mapped on the V,D,J gene segments (VDJ mapped). Then, VDJ mapped and VDJ unmapped reads are aligned against V and J gene segments using Blast in order to identify a VJ recombination (VDJ encompassing reads). Finally D gene segments are identified using Shrimp tool. The output of VDJSeq-Solver represents the list of all identified clones.

ARRest/Interrogate [13] is a web-based interactive application for Ig and TCR (T cell receptor) immunoprofiling. ARRest/Interrogate was developed by CEITEC – Central European Institute of Technology, Masaryk University (Brno, Czech Republic). The application includes four functions: input processing, data selection and filtering, comparative calculation and data visualization. The first function deals with V,D and J gene annotation through IMGT. Genes and alleles are combined with the amino acid sequence of the junction regions to construct IMGT-like clonotypes. Data selection and filtering allow the user to select group of samples depending on several features. The third function (i.e. comparative calculation) can calculate and visualize differences among samples comparing the features (single feature or entire feature type) on the basis of their abundance across the samples. The visualization function allows to visualize the results using several type of graph as bubble charts, heatmaps, bar graph, PCA scatterplots or statistical plots.

ViDJil [14] is an open-source platform for the analysis of high-throughput sequencing data from lymphocytes. ViDJil was developed by Laboratoire d'Informatique Fondamentale de Lille and Inria Lille – Cité scientifique (Villeneuve d'Ascq, France). Vidjil processes deep sequencing data in order to extract V(D)J junctions and gather them into clones for quantification. To quantify the clonotype abundances starting from a set of reads, the method proceeds through a first ultrafast prediction of short string-sequence, overlapping the third complementarity-determining region (CDR3) in order to contain the junction region and part

of the V region and J region of each rearrangement. Each clonotype abundance is then estimated using the number of reads containing the same string-sequence. Finally, it is selected one representative sequence per clone. Vidjil results can be visualized and analyzed through a web application in order to track clonotypes along time in a MRD study.

6 Pilot study analysis by HashClone

Study	Patient	ViDJil Time	HashClone Time
Pilot 1	A	~42m.	~8m.
	B	~44m.	~10m.
	C	~42m.	~8m.
Pilot 2	A	~9m.	~1m.
	B	~25m.	~3m.
	E	~8m.	~1m.

Figure 4: Execution Time

HashClone Execution Times HashClone was executed on a laptop with a 2.80GHz processor (Intel Core i7-2640M) and 7.7GB of memory.

HashClone command lines Pilot 1

Patient A

$bash\ HashClone.sh$ 26 10999997 10999997 1 $\sim /output\ null$ $\sim /input/A-S1.fastq$ $\sim /input/AFU1.fastq$ $\sim /input/AFU2.fastq$ $\sim /input/AFU3.fastq$ $\sim /input/BC.fastq$ $\sim /input/H20.fastq$

Patient B

$bash\ HashClone.sh$ 26 10999997 10999997 1 $\sim /output\ null$ $\sim /input/B-S5.fastq$ $\sim /input/BFU1.fastq$ $\sim /input/BFU2.fastq$ $\sim /input/BFU3.fastq$ $\sim /input/BC.fastq$ $\sim /input/H20.fastq$

Patient C

$bash\ HashClone.sh$ 26 10999997 10999997 1 $\sim /output\ null$ $\sim /input/C-S9.fastq$ $\sim /input/CFU1.fastq$ $\sim /input/CFU2.fastq$ $\sim /input/CFU3.fastq$ $\sim /input/BC.fastq$ $\sim /input/H20.fastq$

Patient D

$bash\ HashClone.sh$ 26 10999997 10999997 1 $\sim /output\ null$ $\sim /input/D-S13.fastq$ $\sim /input/DFU1.fastq$ $\sim /input/DFU2.fastq$ $\sim /input/BC.fastq$ $\sim /input/H20.fastq$

Patient E
bash HashClone.sh 26 10999997 10999997 1 ~ /*output null* ~ /*input*/*E* − *S*17.*fastq*
~ /*input*/*EFU*1.*fastq* ~ /*input*/*EFU*2.*fastq* ~ /*input*/*EFU*3.*fastq* ~ /*input*/*BC*.*fastq*
~ /*input*/*H*20.*fastq*

Pilot 2

Patient A
bash HashClone.sh 26 10999997 10999997 0.001 ~ /*output null* ~ /*input*/*A_S*1.*fastq*
~ /*input*/*A* − *FU*1_*S*6.*fastq* ~ /*input*/*A* − *FU*2_*S*7.*fastq* ~ /*input*/*A* − *FU*3_*S*8.*fastq*
~ /*input*/*BC_S*44.*fastq* ~ /*input*/*HELA_S*45.*fastq*

Patient B
bash HashClone.sh 26 10999997 10999997 0.001 ~ /*output null* ~ /*input*/*B_S*9.*fastq*
~ /*input*/*BFU*1_*S*13.*fastq* ~ /*input*/*BFU*2_*S*14.*fastq* ~ /*input*/*BFU*3_*S*15.*fastq*
~ /*input*/*BFU*4_*S*16.*fastq* ~ /*input*/*B_S*44.*fastq* ~ /*input*/*HELA_S*45.*fastq*

Patient E
bash HashClone.sh 26 10999997 10999997 0.001 ~ /*output null* ~ /*input*/*E_S*35.*fastq*
~ /*input*/*EFU*1_*S*40.*fastq* ~ /*input*/*EFU*2_*S*41.*fastq* ~ /*input*/*EFU*3_*S*42.*fastq*
~ /*input*/*EFU*4_*S*43.*fastq* ~ /*input*/*BC_S*44.*fastq* ~ /*input*/*HELA_S*45.*fastq*

To run HashClone from command line you must type the following command:
bash HashClone.sh k-mer hash_size collision_list_size threshold output_folder mail input_file_1 ..input_file_n

where:

- *k-mer* is the size of k-mers encoded in the hash table. This must be a value between 1 and 32;
- *hash_size* is a (prime) number indicating the size of the hash table. Increasing this value reduces the execution time but increases the memory utilization. Ideally, this value should be close to the number of different k-mers stored in the hash table;
- *collision_list_size* the maximum number of different k-mers that the tool might need to store in the hast table. This parameter is required to optimize the memory utilization;
- *threshold* this value is the threshold used to select *significant k-mers* (τ);
- *output_folder* the folder where the output will be saved;
- *mail* null or a valid mail address where to send information on termination status of the run;
- *input_file_1....input_file_n* the list of input patient files (one for each follow-up in fastq format)

7 HashClone performance on IGH alignment using StandfordS_22

We tested the performance of the IGH alignment implemented in HashClone using the Stanford_S22 dataset. In the paper of Jackson et al. [16] the authors evaluated the performance of seven algorithms handling the thousands of IGH rearrangements in Stanford_S22 dataset to identify the IGHV, IGHD and IGHJ assignments and compare these back to the known genes from the inferred genotype for the subject. The thousands of IGH rearrangements in this dataset allow the individual genotype at the variable gene loci to be inferred.

We modified the Stanford_S22 dataset in order to test the performance of HashClone in terms of the IGHV, IGHD and IGHJ assignments. HashClone identifies 111 clones out of 500 having the percentage of alignment major than 90% for IGHV, IGHJ and IGHD. In the following we reported the data grouped by gene:

IGHV gene and allele correct: **99**
IGHV gene correct but different allelic variant: **12**
IGHV different gene not in the genotype: **0**

IGHJ gene and allele correct: **107**
IGHJ gene correct but a different allelic variant: **4**
IGHJ different gene not in the genotype: **0**

IGHD gene and allele correct: **105**
IGHD gene correct but a different allelic variant: **4**
IGHD different gene not in the genotype: **2**

Figure 5 shows the update version of Table 1 of the Jackson et al paper that reports the percentage of alignments from the Stanford_S22 dataset that were made, by various utilities, to IGHV, IGHD and IGHJ genes and allelic variants absent from the S22 genotype

Utility	IGHV (%)[a]	IGHD (%)[a]	IGHJ (%)[a]	Total (%)[b]
iHMMune-align	3.21 (0.21)	2.21 (1.27)	1.95 (0.0)	7.11
IMGT/VQ+JA	4.90 (0.22)	5.09 (2.81)	1.55 (0.0)	10.87
IgBLAST	3.84 (0.75)	3.96 (2.16)	0.85 (0.0)	8.39
Ab-origin	4.06 (0.22)	7.94 (5.53)	2.53 (0.0)	13.74
JOINSOLVER	6.17 (0.86)	6.93 (4.92)	1.24 (0.0)	7.89
SoDA	2.68 (0.29)	6.82 (6.63)	1.50 (0.0)	10.37
VDJSolver	6.87 (0.48)	1.96 (0.79)	0.71 (0.0)	9.09
HashClone	0 (10.8)	1.8 (5.4)	0 (3.6)	1.8

Figure 5: Performance of the algorithms in IGH detection. [a]Errors involving an incorrect gene, rather than an incorrect allelic variant, are shown in brackets. [b] Percentage of sequences that include an incorrect gene or allele for either the V, D or J.

.

The overall error for HashClone is equal to 1.8% that is the lowest value compared to the

overall error percentages reported by Jackson, ranging between 7.1% (using iHMMune-align algorithm) and 13.7% (using Ab-origin algorithm).

8 How the τ choice affects the algorithm performance

As already highlighted in the Material and Methods section, the choice of an appropriated τ can impact on the capability of HashClone to identify clones; therefore here we report some considerations and suggestions to help the user in this task. First of all, the information associated with the biological experiment (e.g. the dilution factor in case of artificial experiments or an estimation of the residual tumor cells) should be exploited by the user to find an appropriate initial τ value. Then, τ value can be decreased to refine the HashClone solution. Indeed smaller τ values leads to a higher number of *significant k-mers* resulting in more specific clone signatures. For instance, in *Pilot1* the initial τ value, as already stated, can be selected equal to 1 since for each MCL patient, a 10-fold standard curve was prepared diluting diagnostic sample in gDNA pool from five healthy donors. Then, smaller τ values can be chosen without severally influencing the major clone characterization. Instead, values of τ greater than 1 do not make sense due to the design of the experiments. An example is showed in Figure 6 in which the major clone MRD trends obtained decreasing τ from 1 to 0.25 for Patient A are reported and compared with ASO q-PCR result. As expected all these trends are similar and close to the ASO q-PCR trend[1].

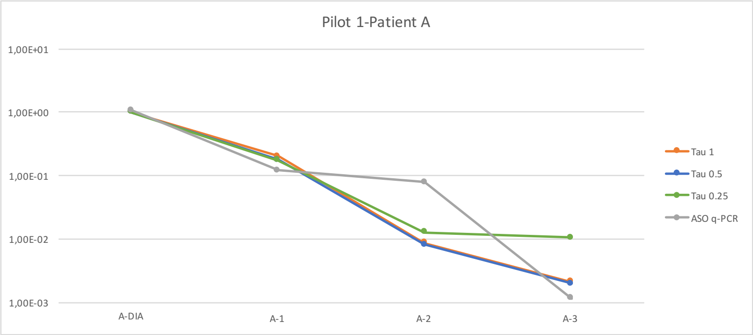

Figure 6: Assesment of τ value. The major clone trends obtained with different τ values on the data of the Patient A of *Pilot1*.

.

[1]Note that a similar results can be obtained for all patients in the two pilots.

References

[1] G Gaidano et al. “p53 mutations in human lymphoid malignancies: association with Burkitt lymphoma and chronic lymphocytic leukemia.” In: *PNAS* 15;88(12) (1991), pp. 5413–7.

[2] C Voena et al. “A novel nested-pcr strategy for the detection of rearranged immunoglobulin heavy-chain genes in b cell tumors”. In: *Leukemia* 11(10) (1997), 1793–1798.

[3] E Alamyar et al. “IMGT/HighV QUEST: the IMGT® web portal for immunoglobulin (IG) or antibody and T cell receptor (TR) analysis from NGS high throughput and deep sequencing”. In: *Immunome Res* 8(1) (2012), p. 26.

[4] VH van der Velden et al. “Detection of minimal residual disease in hematologic malignancies by real-time quantitative PCR: principles, approaches, and laboratory aspects”. In: *Leukemia* 17(6) (2003), pp. 1013–34.

[5] M Ladetto et al. “Real-Time polymerase chain reaction of immunoglobulin rearrangements for quantitative evaluation of minimal residual disease in multiple myeloma”. In: *Biol Blood Marrow Transplant* 6(3) (2000), pp. 241–253.

[6] JJ van Dongen et al. “Design and standardization of PCR primers and protocols for detection of clonal immunoglobulin and T-cell receptor gene recombinations in suspect lymphoproliferations: report of the BIOMED-2 Concerted Action BMH4-CT98-3936”. In: *Leukemia* 17(12) (2003), pp. 2257–2317.

[7] M Kotrova et al. “The predictive strength of next-generation sequencing MRD detection for relapse compared with current methods in childhood ALL”. In: *Blood* 126(8) (2015), pp. 1045–7.

[8] MW Fuellgrabe et al. “High-Throughput, Amplicon-Based Sequencing of the CREBBP Gene as a Tool to Develop a Universal Platform-Independent Assay”. In: *PLoS One* 10(6) (2015).

[9] MM Souto-Carneiro et al. “Characterization of the human Ig heavy chain antigen binding complementarity determining region 3 using a newly developed software algorithm, JOINSOLVER”. In: *Journal of immunology* 172(11) (2004), pp. 6790–802.

[10] BA Gaëta et al. “iHMMune-align: hidden Markov model-based alignment and identification of germline genes in rearranged immunoglobulin gene sequences”. In: *Bioinformatics* 23(13) (2007), pp. 1580–7.

[11] S Munshaw and TB Kepler. “SoDA2: a Hidden Markov Model approach for identification of immunoglobulin rearrangements”. In: *Bioinformatics* 26(7) (2010), pp. 867–72.

[12] G Paciello et al. “VDJSeq-Solver: in silico V(D)J recombination detection tool”. In: *PLoS One* 10(3) (2015), e0118192.

[13] V Bystry et al. “ARResT/Interrogate: an interactive immunoprofiler for IG/TR NGS data.” In: *Bioinformatics* (2016).

[14] M Giraud et al. “Fast multiclonal clusterization of V(D)J recombinations from high-throughput sequencing”. In: *BMC Genomics* 15 (2014), p. 409.

[15] V Giudicelli, D Chaume, and MP Lefranc. “IMGT/GENE-DB: a comprehensive database for human and mouse immunoglobulin and T cell receptor genes”. In: *Nucleic Acids Research* 33 (2005), pp. 256–261.

[16] KJL Jackson et al. “Benchmarking the performance of human antibody gene alignment utilities using a 454 sequence dataset”. In: *Bioinformatics* 26(24) (2010), 3129–3130.
