# Supplementary material for: HashClone: a new tool to quantify the minimal residual disease in B-cell lymphoma from deep sequencing data
Source: BMC Bioinformatics. 2017 Nov 23;18:516. doi: 10.1186/s12859-017-1923-2 (PMC5701356; doi:10.1186/s12859-017-1923-2)
Supplement: Supplementary file 2 — Figure S1. Whole methodology. The whole experimental and computational methodology presented in this paper. (PDF 885 kb) [file 12859_2017_1923_MOESM2_ESM.pdf]

Step 1: Sample processing and gDNA extraction

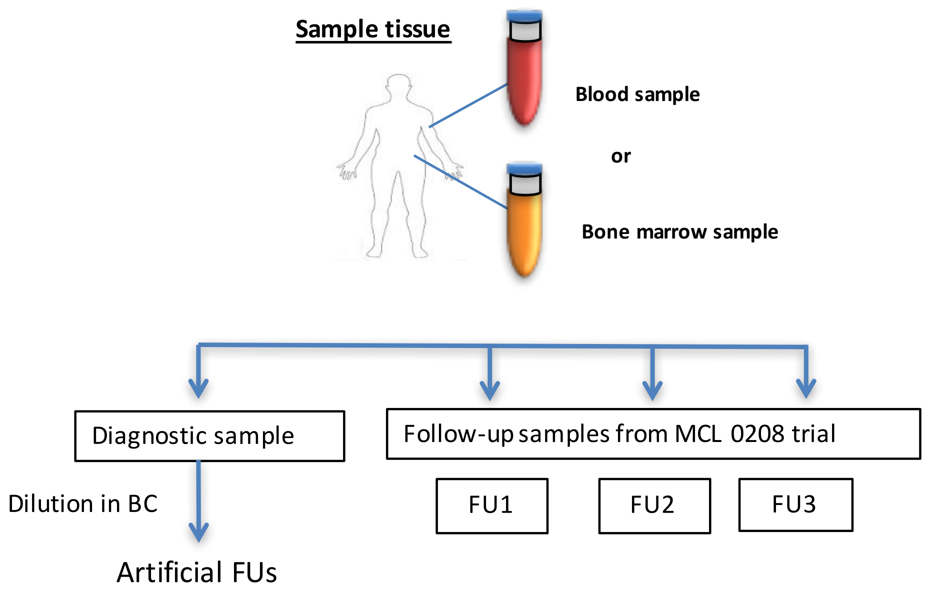

Step 2: Libraries preparation

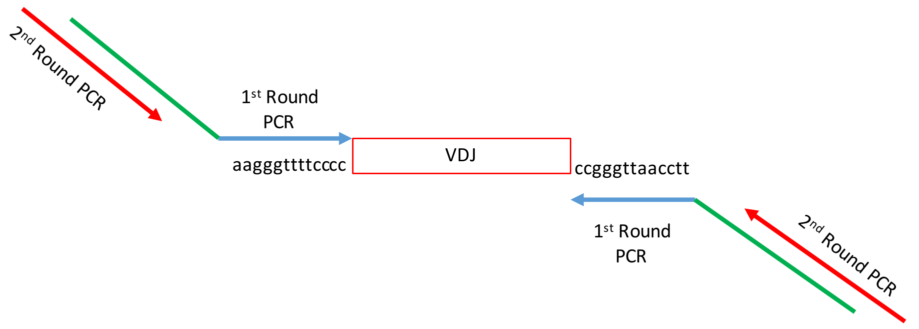

Step 3: Sequencing and FASTQ quality check

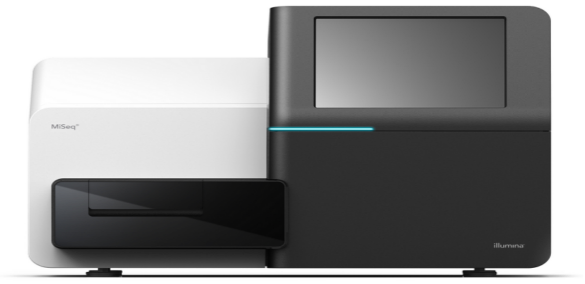

FastQC Report

Step 4: HashClone analysis

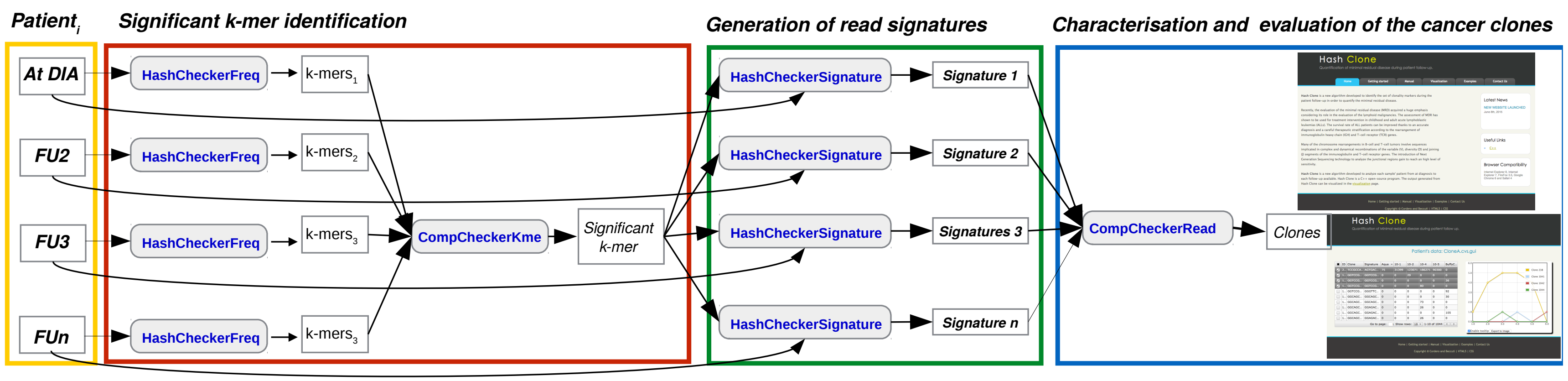

Step 5: Filtered strategy for B-cell clones selection

Phase A:

Clone selection in diagnostic samples with reads frequency higher than 5%

Phase B:

IMGT software: HashClone-VDJ sequences aligment to IGH reference database to check sequence homology.

Step 6: MRD monitoring :

$$\text{FU (Q-PCR value)} = \frac{\text{Dia (Q-PCR value)} \times \text{FU (NGS reads)}}{\text{Dia (NGS reads)}}$$
