# Supplementary material for: HashClone: a new tool to quantify the minimal residual disease in B-cell lymphoma from deep sequencing data
Source: BMC Bioinformatics. 2017 Nov 23;18:516. doi: 10.1186/s12859-017-1923-2 (PMC5701356; doi:10.1186/s12859-017-1923-2)
Supplement: Supplementary file 3 — Table S1. Experimental details. The main features of the samples analyzed. ∗ these samples were analyzed in both Pilot1 and Pilot2. (PDF 29.7 kb) [file 12859_2017_1923_MOESM3_ESM.pdf]

Study (DNA input)	Patients	Samples analyzed (n)	Diagnostic tissue analyzed	Artificial FUs (n)	FUs from clinical trial (n)
Pilot 1 (500 ng)	***A***	4	BM*	3	/
	B	4	PB*	3	/
	C	4	PB	3	/
	D	3	BM	2	/
	E	4	BM*	3	/
Pilot 2 (100 ng)	***A***	4	BM*	/	3
	B	5	PB*	/	4
	E	5	BM*	/	4
