# Supplementary material for: HashClone: a new tool to quantify the minimal residual disease in B-cell lymphoma from deep sequencing data
Source: BMC Bioinformatics. 2017 Nov 23;18:516. doi: 10.1186/s12859-017-1923-2 (PMC5701356; doi:10.1186/s12859-017-1923-2)
Supplement: Supplementary file 4 — Figure S2. Clonotypes quantification by Hashclone. Hashclone identifies an average number of clones equals to 21 in Pilot1 and 32 in Pilot2. In the last column of the table is reported for each major clone the number of reads associates to it with respect to the total number of reads. The same data are also reported for the other clones identified. (PDF 52.7 kb) [file 12859_2017_1923_MOESM4_ESM.pdf]

Study	Patient	Number of clonotypes	Number of read associated with clonotype
Pilot 1	**A**	7	Major clone: 72698/74542 (98%)<br>Other clones: 1844/74542 (2%)
	**B**	32	Major clone:46694/56964(82%)<br>Others:10270/56964(18%)
	**C**	44	Major clone:88674/97018 (91%)<br>Others:8344/97018 (9%)
	**D**	5	Major clone:104908/107808 (97%)<br>Others:2900/107808 (3%)
	**E**	21	Major clone:185456/192947 (96%)<br>Others: 7491/192947 (4%)
	**Average value**	**22**	**Major clone: 93%**<br>**Others: 7%**
Pilot 2	**A**	18	Major clone: 99414/107825 (92%)<br>Others: 8411/107825 (8%)
	**B**	72	Major clone: 169175/231281 (73%)<br>Others: 62106/231281 (27%)
	**E**	5	Major clone: 85027/85603 (99%)<br>Others: 576/85603 (0,1%)
	**Average value**	**32**	**Major clone: 88%**<br>**Others: 12%**

Figure S2 - Clonotypes quantification by Hashclone
