# Supplementary material for: HashClone: a new tool to quantify the minimal residual disease in B-cell lymphoma from deep sequencing data
Source: BMC Bioinformatics. 2017 Nov 23;18:516. doi: 10.1186/s12859-017-1923-2 (PMC5701356; doi:10.1186/s12859-017-1923-2)
Supplement: Supplementary file 5 — Figure S3. Clonotypes identification by ViDJil. The clonotypes identified by ViDJil in Pilot1 and Pilot2 are reported in the third column. In the fourth column are reported the clones passed the Phase A while in the fifth column there are the number of clones passed the Phase B. (PDF 46.4 kb) [file 12859_2017_1923_MOESM5_ESM.pdf]

Study	Patient (only diagnosis samples)	Clonotype identified	Phase A<br>Clonotype with frequency > 100	Phase B<br>Clonotype associated with VDJ
Pilot 1	**A**	159	49	30
	**B**	171	51	31
	**C**	163	51	43
	**D**	129	50	42
	**E**	165	52	38
	**Average value**	**157**	**51**	**37**
Pilot 2	**A**	138	43	34
	**B**	196	72	50
	**E**	108	45	39
	**Average value**	**114**	**53**	**41**

Figure S3 - Clonotypes identification by ViDJil
