# Supplementary material for: HashClone: a new tool to quantify the minimal residual disease in B-cell lymphoma from deep sequencing data
Source: BMC Bioinformatics. 2017 Nov 23;18:516. doi: 10.1186/s12859-017-1923-2 (PMC5701356; doi:10.1186/s12859-017-1923-2)
Supplement: Supplementary file 6 — Figure S4. Clonotypes quantification by ViDJil. ViDJil identifies an average number of clones equals to 37 in Pilot1 while in Pilot2 it does not identified any clonotypes. In the last column of the table is reported for each major clone the number of reads associates to it with respect to the total number of reads. The same data are also reported for the other clones identified. (PDF 51.9 kb) [file 12859_2017_1923_MOESM6_ESM.pdf]

Study	Patient	Number of clonotype	Number of read associated with Clonotype
Pilot 1	**A**	30	Major clone: 83974/86619 (97%)<br>Other clones: 2645/86619 (3%)
	**B**	31	Major clone: 58620/65302 (90%)<br>Others: 6682/65302 (10%)
	**C**	43	Major clone: 108386/121593 (98%)<br>Others: 13207/121593 (2%)
	**D**	42	Major clone: 180075/183559 (98%)<br>Others: 3484/183559 (2%)
	**E**	38	Major clone: 217524/217917(99%)<br>Others: 393/217917 (1%)
	**Average value**	**37**	**Major clone: 96%**<br>**Others: 4%**
Pilot 2	**A**	0	/
	**B**	0	/
	**E**	0	/

Figure S4 - Clonotypes quantification by ViDJil
