# Supplementary material for: HashClone: a new tool to quantify the minimal residual disease in B-cell lymphoma from deep sequencing data
Source: BMC Bioinformatics. 2017 Nov 23;18:516. doi: 10.1186/s12859-017-1923-2 (PMC5701356; doi:10.1186/s12859-017-1923-2)
Supplement: Supplementary file 7 — Figure S5. ViDJil and Sanger Sequence comparison. Nucleotide alignments between the complementary region 3 sequences (CDR3, indicated in bold and underline) Sanger sequence and the sequence identified by ViDJil. (PDF 60.9 kb) [file 12859_2017_1923_MOESM7_ESM.pdf]

Study	Patient	CDR3 Sanger Sequence	CDR3 Vidjil sequence	Homology
Pilot 1	A	GCGAGAGA**TCCA**GGGTATAGCAGTGGCTGGAA C**CTGGGA**TACTACTACTACGGTATGGACGTC	GCGAGAGA**TCCA**GGGTATAGCAGTGGCTGGAA C**CTGGGA**TACTACTACTACGGTATGGACGTC	100%(63/63nt)
	B	TGTGCGAGAAGCAATTTTGGAGTGG**TCTAAAT** **TACAT**GGACGTCT	TGTGC**NN**GAA**T**CAATTTTGGAGTGG**TCTAAAT** **TACAT**GGACGTCT	93%(42/45nt)
	C	CGAGAGA**TTACACAGCCCC**GGGTATAGCAGAA CCAGGC**CCC**T	CGAGAGA**TTACACAGCCCC**GGGTATAGCAGAA CCAGGC**CCC**T	100%(42/42)
	D	TGCGAGAGG**CGCGA**ATAACTGGAAC**CCCA**TTG ACTA	TGCGAGAGG**CGCGA**ATAACTGGAAC**CCCA**TTG ACTA	100%(36/36nt)
	E	GCGA**CCCAGCGAA**ATTACGATATTTTGACCGG **G**TTTGACTACT	GCGA**CCCAGCGAA**ATTACGATATTTTGACCGG **G**TTTGACTACT	100%(43/43nt)
Pilot 2	A	GCGAGAGA**TCCA**GGGTATAGCAGTGGCTGGAA C**CTGGGA**TACTACTACTACGG	CACGGNTGTGTATTNNTGTGCNNGNANNNNNG NGTNTANNNGTGNNNGNNANCNNGNANNCTNN	0%(0/59nt)
	B	TGTGCGAGAAGCAATTTTGGAGTGG**TCTAAAT** **TACAT**GGACGTCT	GNAAAACGACGGCCAGTTGGATCCGTCAGCCC CCAGGGAAGGTCACCGTCTCCTCAGGTAAGCC CTATAGTGAGTCGTATTA	0%(0/45nt)
	E	GCGA**CCCAGCGAA**ATTACGATATTTTGACCGG **G**TTTGACTACT	GNAAAACGACGGCCAGTTTGGGTGCGACAGGC CCCTGGACAAGGGCTTGAGTGGNTGGGATGGA	0%(0/43nt)

Figure S5 - ViDJil and Sanger Sequence comparison
